# Different contributions of primary motor cortex, reticular formation, and spinal cord to fractionated muscle activation

**DOI:** 10.1152/jn.00672.2017

**Published:** 2017-10-18

**Authors:** Boubker Zaaimi, Lauren R. Dean, Stuart N. Baker

**Affiliations:** Institute of Neuroscience, Newcastle University, Newcastle upon Tyne, United Kingdom

**Keywords:** electromyogram, fractionation, motor cortex, reticular formation, spinal cord, synergy

## Abstract

Coordinated movement requires patterned activation of muscles. In this study, we examined differences in selective activation of primate upper limb muscles by cortical and subcortical regions. Five macaque monkeys were trained to perform a reach and grasp task, and electromyogram (EMG) was recorded from 10 to 24 muscles while weak single-pulse stimuli were delivered through microelectrodes inserted in the motor cortex (M1), reticular formation (RF), or cervical spinal cord (SC). Stimulus intensity was adjusted to a level just above threshold. Stimulus-evoked effects were assessed from averages of rectified EMG. M1, RF, and SC activated 1.5 ± 0.9, 1.9 ± 0.8, and 2.5 ± 1.6 muscles per site (means ± SD); only M1 and SC differed significantly. In between recording sessions, natural muscle activity in the home cage was recorded using a miniature data logger. A novel analysis assessed how well natural activity could be reconstructed by stimulus-evoked responses. This provided two measures: normalized vector length *L*, reflecting how closely aligned natural and stimulus-evoked activity were, and normalized residual *R*, measuring the fraction of natural activity not reachable using stimulus-evoked patterns. Average values for M1, RF, and SC were *L* = 119.1 ± 9.6, 105.9 ± 6.2, and 109.3 ± 8.4% and *R* = 50.3 ± 4.9, 56.4 ± 3.5, and 51.5 ± 4.8%, respectively. RF was significantly different from M1 and SC on both measurements. RF is thus able to generate an approximation to the motor output with less activation than required by M1 and SC, but M1 and SC are more precise in reaching the exact activation pattern required. Cortical, brainstem, and spinal centers likely play distinct roles, as they cooperate to generate voluntary movements.

**NEW & NOTEWORTHY** Brainstem reticular formation, primary motor cortex, and cervical spinal cord intermediate zone can all activate primate upper limb muscles. However, brainstem output is more efficient but less precise in producing natural patterns of motor output than motor cortex or spinal cord. We suggest that gross muscle synergies from the reticular formation are sculpted and refined by motor cortex and spinal circuits to reach the finely fractionated output characteristic of dexterous primate upper limb movements.

## INTRODUCTION

Controlling movement requires that a large number of muscles are activated in the correct pattern. The complexity of this problem has led to suggestions that the nervous system uses dimensional reduction, controlling a small number of functional synergies rather than every muscle independently (Saltiel et al. 2001). Different versions of this idea defined either spatial (a set of muscles, activated in fixed ratios) or spatiotemporal (time-varying templates of muscle coactivations) synergies (Overduin et al. 2015).

To investigate the idea of dimensional reduction, Santello et al. (1998) recorded joint angles of the human hand during a wide range of grasps and abstract gestures and subjected the recordings to principal component analysis. In this highly diverse data set, 85% of the total variance could be explained by just two components, which seems to support dimensional reduction at least for hand function. Overduin et al. (2012) showed that stimulation of the motor cortex in macaque monkeys produced muscle activations well represented by a small number of synergies, suggesting that synergies had a basis in the underlying neural representation.

Some recent work has questioned the importance of synergies in the control of the human hand. When subjects generate isometric forces with the index finger, the variance profile of the force and the coactivation of different muscles fit better with a flexible control scheme in which muscles can be addressed individually, rather than in fixed synergies (Kutch et al. 2008; Valero-Cuevas et al. 2009). Even if synergies can be detected, it is not necessarily the case that they are the result of deliberate control strategies. Kutch and Valero-Cuevas (2012) used a cadaveric hand with tendons actuated by motors to perform an index finger movement task. For successful task performance, biomechanical constraints led to obligate correlations in muscle activities, which would appear as synergies in a typical analysis, even though no such constraints were imposed by the controller. Finally, dimensional reduction seems to be less apparent if a wider range of movements is considered. When recording joint angles with a portable system during everyday life, Ingram et al. (2008) found that only 60% of the total variance could be explained by two principal components; 10 components (out of a possible total of 19) were required to explain 95%. It is possible that the apparent dominance of two synergies found by Santello et al. (1998) was thus an artifact of artificially constraining hand function to a set of laboratory-based tasks. Interestingly, although the remaining components contributed little to the overall variance in the study by Santello et al. (1998), the magnitude of these higher components critically distinguished different grasps. This led to the suggestion that a more selective activation of muscles is superimposed onto low-dimensional synergies, which permits fine fractionation of digit movement.

Much previous work points to the importance of the corticospinal tract in mediating fine fractionated finger movements. Following a lesion of the corticospinal tract at the medulla or at spinal segment C2, monkeys show a permanent impairment of independent finger movements (Alstermark et al. 2011; Lawrence and Kuypers 1968). Similar difficulties with isolated finger movements are seen in stroke patients after corticospinal damage (Lang and Schieber 2003; Raghavan et al. 2006). Infant macaques develop corticospinal projections to motoneuron pools in the cervical cord around the time that they first start grooming, a behavior that is impossible without fine hand control (Armand et al. 1997). Monkeys that have strong cortico-motoneuronal connections have more dexterous hand function than those with only weak direct connections to motoneurons (Bortoff and Strick 1993; Maier et al. 1997).

There is an increasing realization that subcortical systems make an important contribution to the control of the primate hand. As well as the corticospinal tract, the reticulospinal tract provides inputs to motoneurons innervating forearm and intrinsic hand muscles (Riddle et al. 2009), and neurons in the reticular formation (RF) modulate their activity during fine finger movements (Soteropoulos et al. 2012). Propriospinal interneurons located at the C3–C4 spinal segments receive corticospinal (Isa et al. 2006) and reticulospinal input (Illert et al. 1981), project to motoneurons innervating the hand and forearm, and can partially mediate fine finger control (Kinoshita et al. 2012; Sasaki et al. 2004). Within the cervical enlargement, many segmental interneurons also have corticospinal and reticulospinal input (Riddle and Baker 2010), provide inputs to motoneurons controlling the hand, and modulate their activity during fine grasp (Takei and Seki 2010).

Although brainstem and spinal circuits undoubtedly contribute to hand control, they seem to have different roles (Baker et al. 2015). Identified cortico-motoneuronal cells modulate their firing strongly with a precision grip, but only weakly with a power grip, even when the muscle facilitated by their corticospinal projection is more active in a power grip (Muir and Lemon 1983). This suggests a role limited to fine, but not gross, hand function. The firing rate of neurons in primary motor cortex (M1) correlates better with individual finger joint angles than principal component scores derived from those joint angles (Kirsch et al. 2014). We know that cortico-motoneuronal cells and spinal cord interneurons facilitate small groups of motoneuron pools (Buys et al. 1986, Takei and Seki 2010), whereas reticulospinal axons branch extensively within the spinal cord, and hence presumably coactivate a large group of muscles (Peterson et al. 1975). This might suggest that brainstem pathways provide the neural origin for the small numbers of synergies often detected in principal component analysis, whereas cortical (and possibly spinal) circuits permit more flexible control of individual muscles.

In patients who have lost corticospinal pathways following a stroke, principal component analysis can reveal a similar synergy structure for movements with the more affected and less affected arm (Cheung et al. 2009, although this is not a universal finding, Roh et al. 2013). This is expected if surviving brainstem pathways continue to contribute a gross synergy structure to movements even after recovery from damage. Indeed, the excessive and obligate coactivation of fixed muscle groups is a defining feature of movements in recovered stroke patients (Dewald et al. 1995; Kamper and Rymer 2000).

To date, no studies have made a direct comparison between the outputs of M1, RF, and the spinal cord and whether these would better support fixed synergies or more flexible, independent activation of muscles in the upper limb. Overduin et al. (2012) used long trains of intracortical microstimulation to M1 to elicit movements. Nonnegative matrix factorization of the resulting muscle activity revealed a similar underlying synergy structure, as extracted during performance of natural grasp movements. Activity generated by such long trains of stimuli may spread to distant cortical and subcortical sites, and therefore, it is unclear whether the synergies detected originated within M1 or in its output targets. Output patterns have been mapped using more focal single-pulse stimuli from M1 (Park et al. 2004), lateral (Boudrias et al. 2010b), and mesial (Boudrias et al. 2010a) premotor cortex, red nucleus (Belhaj-Saïf et al. 1998), reticular formation (Davidson and Buford 2006, Soteropoulos et al. 2012), and spinal cord (Moritz et al. 2007) and also by examining the post-spike facilitation seen in spike-triggered averages of EMG (Buys et al. 1986; Cheney and Fetz 1980; Davidson et al. 2007b; Mewes and Cheney 1991; Takei and Seki 2010). These studies have reported generally overlapping ranges of numbers of muscles activated per site and have not considered how the patterns of coactivated muscles relate to patterns seen in natural activity.

In this study, we gathered an extensive data set of responses in upper limb muscles to single-pulse stimulation in M1, RF, and the cervical enlargement of the spinal cord (SC) in macaque monkeys. Activity in the same muscles was recorded during free behavior in the home cage, using a novel miniature data logger. Using a new analysis that tried to reconstruct the natural activity patterns as linear combinations of the stimulus-evoked patterns, we found significant differences between the three regions. RF outputs were better matched to the natural activity, allowing reconstruction of natural patterns that were simpler than using outputs from either M1 or SC. However, the residuals, the parts of natural activity that could not be reconstructed from stimulus-evoked responses, were significantly larger for RF than for M1 or SC. Our results support the idea that RF represents approximate patterns of muscle coactivation, whereas M1 and SC permit more nuanced, selective activity, which underlies the fine fractionated movements so important to the primate upper limb.

## METHODS

Experiments were conducted in five female macaca mulatta monkeys (weight: 5.7–8 kg), which are coded *Sa*, *Su*, *P*, *T*, and *U* in this report. All animal procedures were carried out under appropriate licenses issued by the UK Home Office under the Animals (Scientific Procedures) Act 1986 and were approved by the Animal Welfare and Ethical Review Board of Newcastle University.

### 

#### Behavioral task.

**Fig. 1.**
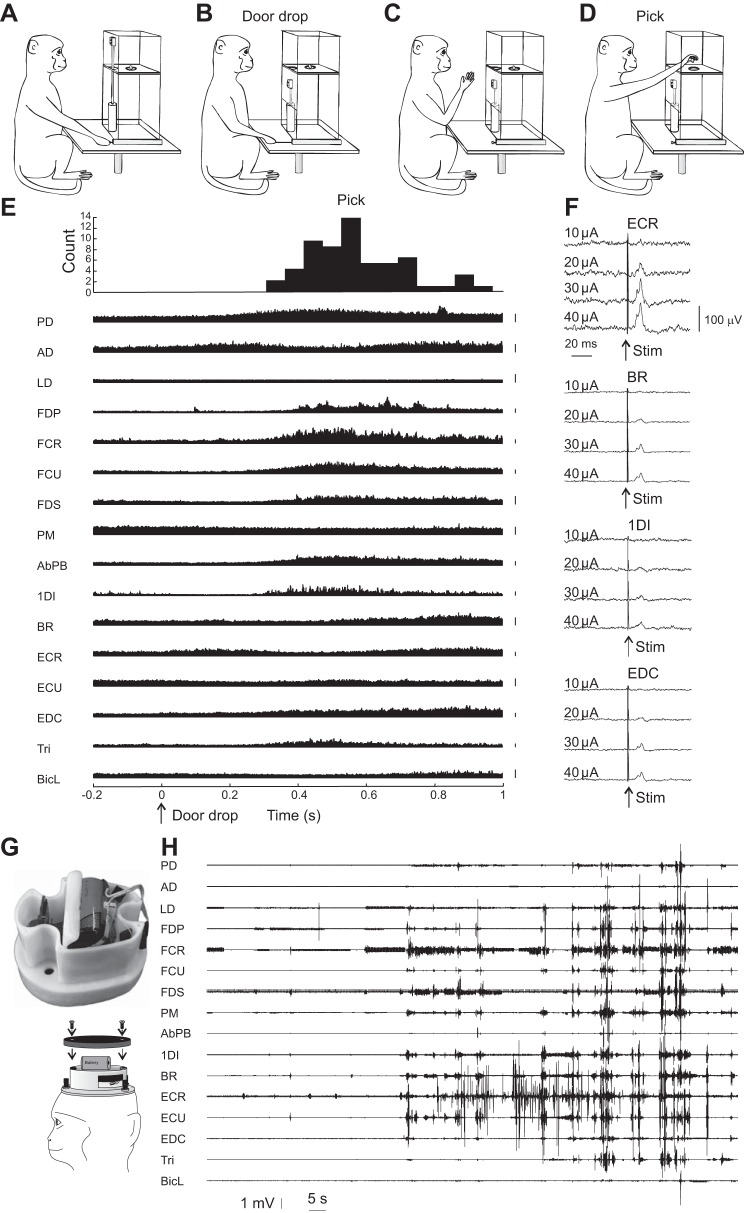
Illustration of methods. *A*–*D*: behavioral task. The monkey grasped (*A*) and pulled (*B*) a lever, which caused a clear plastic door to drop, revealing a food well. The monkey then reached into the well (*C*) and retrieved the food reward (“pick” event, detected by crossing an infrared beam; *D*). *E*: averaged rectified electromyogram (EMG) aligned to the “door drop” event of the task. Histogram at the *top* shows the distribution of the pick event relative to door drop. Vertical calibration bars to the *right* of each trace represent 50 µV. *Monkey P*, *n* = 81 trials. *F*: example responses to motor cortex (M1) stimulation (Stim) in *monkey T*. Each trace shows an average of rectified EMG from the muscle and stimulus indicated for *n* = 800 sweeps. Threshold intensity was determined as 20 µA for this site. *G*: photograph of data logger inside custom case and drawing illustrating how the data logger attached to the head implant on the monkey. *H*: example recording of natural activity from the data logger from *monkey P*. PD, posterior deltoid; AD, anterior deltoid; LD, latissimus dorsi; FDP, flexor digitorum profundus; FCR, flexor carpi radialis; FCU, flexor carpi ulnaris; FDS, flexor digitorum superficialis; PM, pectoralis major; AbPB, abductor pollicis brevis; 1DI, first dorsal interosseous; BR, brachioradialis; ECR, extensor carpi radialis; ECU, extensor carpi ulnaris; EDC, extensor digitorum communis; Tri, triceps; BicL, long head of biceps.

The monkeys were trained to perform the food retrieval task used in Riddle et al. (2009) and illustrated in Fig. 1, *A*–*D*. A trial was initiated by the experimenter baiting a food well with a small piece of food. The animal then pulled a lever (Fig. 1*B*), which activated a pneumatic cylinder attached to a clear plastic door, which prevented access to the food well (Fig. 1*C*). The dropping of the door was the cue for the animal to reach into the well and retrieve the food (Fig. 1*D*), which was then carried to the mouth. This task was chosen because it is relatively straightforward to learn but causes activation of a wide range of proximal and distal muscles in the upper limb (Fig. 1*E*).

#### Surgical implant.

Once animals were trained on the task, they underwent two implant surgeries, using a full aseptic technique. Initial sedation used intramuscular injections of ketamine (10 mg/kg). An intravenous line was then placed and anesthesia induced using propofol (2.3 mg/kg). The animal was then intubated and anesthesia maintained using inhalation of sevoflurane (∼2% in 100% O_2_) with an intravenous infusion of alfentanil (0.2–0.3 μg·kg^−1^·min^−1^). Intravenous methypregnisolone (loading dose of 30 mg/kg, followed by continual infusion of 5.4 mg·kg^−1^·h^−1^) was used to reduce edema together with a subcutaneous injection of meloxicam (0.3 mg/kg). Dehydration was prevented by an infusion of Hartmann’s solution (to give a total fluid infusion rate, including drug solutions of 5–10 ml·kg^−1^·h^−1^). The animal was kept warm using a thermostatically controlled heating blanket, and a separate source of air warmed to 37°C. A positive pressure ventilator was used throughout to ensure adequate ventilation. Monitoring during surgery included pulse oximetry, heart rate, blood pressure (measured using a cuff on the leg), core and peripheral temperature, and end-tidal CO_2_. Drug doses were adjusted to produce a stable plane of deep general anesthesia.

The first surgery placed electrodes for electromyogram (EMG) recording. Pairs of insulated stainless-steel wires, bared for 1–2 mm at their tips, were inserted into upper limb muscles and secured using silk sutures. The wires were tunneled subcutaneously to connectors. At the end of the surgery, these connectors were sutured into pouches made from sheets of silicone rubber. The pouches were placed under the skin of the back, all wounds were closed, and the animal was allowed to recover from the anesthetic.

Several weeks later, a second surgery implanted a headpiece to permit atraumatic head fixation. The headpiece was produced from carbon fiber-reinforced TekaPEEK and was machined to fit a digital model of the skull produced from a structural MRI scan. The headpiece was attached to the skull using the system of disks and expanding bolt assemblies described by Lemon (1984). Three bolts protruded from the headpiece to allow head fixation during recording sessions and allowed attachment of the data logger (see below). The EMG connectors were retrieved from the back pouches in this surgery, tunneled to the head, and attached to the headpiece. Recording chambers were also attached to the headpiece and centered over craniotomies, which permitted microelectrode penetrations either to the brainstem or motor cortex.

#### Recording sessions in the awake state.

After recovery from the surgery was complete, daily recording sessions began. Tungsten microelectrodes (300-μm shaft diameter, 3-μm tip diameter, 0.1-MΩ impedance; Microprobe, Gaithersburg, MD) were inserted through the craniotomy to target the primary motor cortex (M1) or the pontomedullary reticular formation (RF). Electrodes were advanced while trains of intracranial microstimuli (ICMS) were delivered (13–18 biphasic pulses, 0.2 ms/phase; isolated stimulator model 2100; AM Systems, Sequim, WA) until a site that evoked upper limb movements was located.

Single-pulse ICMS was then delivered to the chosen site (biphasic stimuli, 0.2 ms/phase; repetition rate of 9 Hz) while the animal performed the behavioral task. EMG signals from the implanted electrodes were amplified (gain: 200–5,000; filter bandpass: 30 Hz to 2 kHz) and sampled to disk (5-k samples/s) along with task and stimulus markers using data acquisition cards (National Instruments, Austin, TX) and custom software. In *monkeys Sa* and *U*, a single intensity of stimulus was delivered, chosen to be close to the threshold determined for an overt twitch response to the ICMS train, and 50 trials of the task were recorded. Task performance was then paused, and data were immediately analyzed using a custom script written in the MATLAB environment. This computed averages of rectified EMG and determined whether there was a significant response in any muscle. The intensity was then increased or reduced, and a further recording was gathered. At each site, we aimed to gather a data set at the threshold intensity, which was defined as the intensity T where a response was produced, but no response was produced at 5 µA below T. This required that we gather a minimum of two data sets, at T and T-5 µA, although typically more were required to find the correct choice of intensity T. On some recording days where threshold for the initial site was identified quickly, we moved the electrode to a new site and gathered further data there. Such sites were separated by a depth of 0.4–1.9 mm.

In *monkeys P* and *T*, an alternative approach was used for threshold identification. Stimuli were typically delivered at 10, 20, 30, and 40 µA, in a pseudorandom sequence, using a computer-controlled stimulator (DS4; Digitimer, Welwyn Garden City, UK); recordings were made for 75 trials of the task. In some recordings, a further data set with higher (20, 30, 40, and 50 µA) or lower (5, 10, 15, and 20 µA) intensities was gathered to characterize the threshold better. The lowest intensity that gave a significant EMG response in at least one muscle was denoted as the threshold. Example responses are shown in Fig. 1*F*.

#### Recording natural patterns of EMG activity.

At the end of some recording sessions, the animal was fitted with a miniature custom-built data logger. This was based around a dsPIC33FJ128MC804 microcontroller (Microchip, Chandler, AZ) and two 16-channel differential amplifiers (RHA2216; Intan Technologies, Los Angeles, CA). Up to 32 channels of bipolar EMG were recorded by connecting this custom circuit to the connectors on the headpiece. EMG was amplified (gain: 200; filter: 20 Hz to 1 kHz) and sampled continually at 1,024 or 1,250 samples/channel with 12-bit resolution. In our past experience with similar EMG recordings, we have found minimal power above 500 Hz; aliasing of frequencies above the Nyquist limit was thus unlikely to distort the recorded signals appreciably. Data were stored on a microSD card (32-GB storage capacity), using the FAT32 filing system. The circuit was powered by a lithium ion battery (capacity: 8.5 Ah; Tadiran part no. SL2770/T). The entire system was mounted in a custom enclosure that fitted onto the headpiece (Fig. 1*G*). The monkey was then returned to the home cage, which comprised large enclosures with floor area of 17 m^2^ and was 2.4 m high. The cage was fitted with swings, ropes, and perches, which the animals used to climb and move around the whole three-dimensional space. The floor was covered with shavings, into which seeds were placed to encourage foraging. Monkeys lived in compatible pairs and spent considerable periods of time interacting with their cage mate.

At the end of the data logger recording, the monkey was again brought to the laboratory, and the SD card was retrieved for download to a computer. An example of an EMG recording is shown in Fig. 1*H*.

#### Terminal experiment.

Once recordings in the awake state were completed, an experiment under terminal anesthesia was carried out to record responses to spinal cord stimulation. The monkey was sedated by intramuscular injection of ketamine (10 mg/kg), and anesthesia was induced by intravenous injection of propofol (2.3 mg/kg). Surgical procedures were performed under sevoflurane anesthesia (1–2% in 100% O_2_) with a continuous intravenous infusion of alfentanil (0.2–0.3 μg·kg^−1^·min^−1^). Methypregnisolone and Hartmann’s infusions were given as described above. Initial surgical preparation included a tracheotomy, and central arterial and venous lines were inserted via the major vessels of the neck. The bladder was catheterized. A laminectomy was then performed to expose spinal segments C3-T1. The animal was mounted in a spinal frame. Sevofurane anesthesia was then replaced by intravenous infusion of ketamine (6 mg·kg^−1^·h^−1^), midazolam (0.3–0.6 mg·kg^−1^·h^−1^), and alfentanil (same dose as above). Throughout the procedure, the animal was kept warm by a thermostatically controlled blanket and a separate system using warm air. A positive-pressure ventilator ensured adequate ventilation. Monitoring included pulse oximetry, heart rate, arterial and venous blood pressure (measured via the central lines), core and peripheral temperature, and end-tidal CO_2_. Rapid changes in heart rate or blood pressure following noxious stimuli or more gradual rising trends were taken as a sign of waning anesthesia, and infusion rates were adjusted accordingly.

The implanted EMG electrodes were connected to recording amplifiers. Microelectrode penetrations (electrodes as described above) were then made into the spinal cord between segments C3 and T1. These segments were chosen, as they contain segmental and propriospinal interneurons that control motoneurons innervating the upper limb. The penetration depth targeted the intermediate zone, where such interneurons are located. EMG responses to single-pulse microstimulation were then recorded as above.

At the end of the recordings (which typically lasted 15–34 h), anesthesia was deepened to a lethal level, and the animal was perfused through the heart with phosphate-buffered saline, followed by formalin fixative.

Structural MRI was used to reconstruct the electrode recording sites. The acquisition of the MRI data was described in detail in Baker et al. (1999). In brief, before implant, the monkey was sedated with ketamine (10 mg/kg) and intubated and anesthesia continued by inhalation of isoflurane (2–2.5%). Ventilation, blood oxygen saturation, and heart rate were continuously monitored. The head was fixed into a plastic stereotaxic frame, and T1- and T2-weighted images were acquired. Brain image coordinates were transformed from the scanner arbitrary axes to stereotaxic space (Baker et al. 1999). The location of electrode penetrations was measured each day relative to a fiducial mark on the recording chamber. Using the stereotaxic coordinates of the chamber mark (measured during the implant surgery), we could calculate the location of the electrode tip in stereotaxic coordinates. To reconstruct the penetrations in the reticular formation, the stereotaxic coordinates of the anterior and posterior commissures were identified on the MRI for each monkey. The penetration sites in the reticular formation were then adjusted to bicommissural coordinates and projected on an atlas template (Martin and Bowden 1996).

#### Analysis.

The first stage of analysis involved compiling stimulus-triggered averages of rectified EMG. The EMG was analyzed using a custom program (MatLab; The Mathworks, Natick, MA) to find any significant muscle responses. To determine significant responses for any one site, the program compiled an average rectified trace for each muscle across stimuli within a window of 100 ms before to 150 ms after the stimulus pulse. The 100 ms of EMG before the pulse was defined as the baseline period. The program then searched for any points that exceeded the mean + 3 SD of the baseline during the period 5–25 ms after the stimulus pulse. The average EMG in the response window was measured on single sweeps together with the average from the same duration window preceding the stimulus pulse. A paired *t*-test measured if there was a significant difference between response and baseline periods for that muscle (*P* < 0.05). This was repeated for each muscle for a particular site. Subsequent analysis used these vectors of response amplitude in each recorded EMG, with zeros denoting no significant response. Vectors with no significant responses (i.e., following a stimulus which was subthreshold for all muscles) were excluded.

For recordings of natural activity using the data logger, some EMGs had an artifactual contamination by electrocardiogram (ECG). We have found that this is unavoidable when recordings are taken of more proximal muscles near to the chest. EMGs were first processed using an algorithm that detected QRS complexes in one channel in which they were especially prominent. These were used to generate average templates of the artifact at this time for each other channel; the template was then subtracted from each sweep.

Sections of recording between 7 PM and 7 AM were excluded from further analysis. This was the time when the lights in our housing unit went out, and the animals typically slept; any movements over this time were likely to be involuntary postural adjustments during sleep.

Daytime EMGs with the ECG artifact removed were then high-pass filtered at 30 Hz to remove movement artifacts, rectified, low-pass filtered at 10 Hz to extract the modulation envelope, and downsampled to 50 samples/s. For each channel, a region where no EMG activity was present was chosen by eye from a display of the first 200 s of data. The maximum of this region was used as a threshold, with values below it taken to denote inactivity on that channel. At each sample point, if all channels were inactive (Boolean logical and operation), that sample point was excluded from further analysis. The result of this process was a series of EMG vectors denoting activity in each muscle during natural movements in the home cage.

#### Principal component analysis.

One problem with making quantitative comparisons of fractionation between different areas and animals was the varying number of EMGs available in each case. To permit a valid comparison, ten muscles were chosen in each animal (see Table 1). Each EMG was divided by the standard deviation of that recording in the natural activity recorded by the data logger. Principal component analysis (Dean and Baker 2017) was then performed on the vectors of muscle responses following stimulation and also on the vectors of natural activity recorded in the home cage and activity recorded during performance of the behavioral task in the laboratory. Responses to all available stimulus intensities were used for this analysis. The cumulative percentage of variance explained was plotted vs. the number of principal components.

**Table 1.** Database available for analysis in this report

Monkey ID	Data Logger Recording Duration, h	No. of EMGs	List of EMGs	Brain/SC area	No. of Sites Stimulated	Total No. of Stimulus Responses
*P*	20.0/22.9	16	LD AD PD PM BicS Tri BR EDC ECU ECR FDS FCU FCR FDP 1DI AbPB	RF	23	50
		14	LD PD BicS Tri BR EDC ECU ECR FDS FCU FCR FDP 1DI AbPB	SC	78	207
*Sa*	6.9/11.3	19	LD Cor BicL BicS Bra BR EDC ED2,3 ED4,5 FDS FPL FCU PT FDP FCR PL AbPB ADM 1DI	RF	12	47
				SC	97	335
*T*	28.3/40.3	16	LD PM PD AD BicS Tri BR EDC ECU ECR FDS FDP FCR FCU 1DI AbPB	M1	48	106
*U*	3.3/3.4	10	LD PD BicS BR ECU ECR EDC FCU FDS FDP	M1	20	98
*Su*	Not available	24	LD AD PD Tri Bra Ter Cor BicS BicL BR PT EPL EPB ED2,3 FDS FDP FCR PL ECU FCU APL AbPB 1DI ADM	SC	105	366

M1, primary motor cortex; SC, spinal cord; RF, reticular formation; EMG, electromyogram; LD, latissimus dorsi; PM, pectoralis major; PD, posterior deltoid; AD, anterior deltoid; Cor, coracobrachialis; Ter, teres major; BicS, short head of biceps; BicL, long head of biceps; BR, brachioradialis; Bra, brachialis; Tri, triceps; FDS, flexor digitorum superficialis; FDP, flexor digitorum profundus; FCU, flexor carpi ulnaris; FCR, flexor carpi radialis; PL, palmaris longus; FPL, flexor pollicis longus; EDC, extensor digitorum communis; ED2,3, extensor of digits 2,3; ED4,5, extensor of digits 4,5; EPL, extensor pollicis longus; EPB, extensor pollicis brevis; ECR, extensor carpi radialis; ECU, extensor carpi ulnaris; PT, pronator teres; APL, abductor pollicis longus; AbPB, abductor pollicis brevis; 1DI, first dorsal interosseous; ADM, abductor digiti minimi. “Data logger recording duration” shows the duration of daytime recordings that were assessed as active (see methods) and the total duration taken during daytime hours. “No. of EMGs” indicates channels recorded in that animal, which had at least 1 stimulus-evoked response. Underlined channels in the list of EMGs indicate the subset of 10 channels used in some parts of the analysis. “No. of sites” indicates unique electrode positions, at which responses at threshold were identified. “Total no. of stimulus responses” indicates all stimuli intensities tested that gave a response in at least 1 muscle. The following gives a list of muscle abbreviations, which are grouped according to the classification of muscle groups used in Figs. 4 and 5. Shoulder: LD, PM, PD, AD, Cor, and Ter. Elbow flexors: BicS, BicL, BR, and Bra. Elbow extensor: Tri. Forearm flexors: FDS, FDP, FCU, FCR, PL, and FPL. Forearm extensors: EDC, ED2,3, ED4,5, EPL, EPB, ECR, and ECU. Other forearm: PT and APL. Intrinsic hand: AbPB, 1DI, and ADM.

#### Space similarity analysis.

Principal component analysis reveals how efficiently patterns of muscle activity may be expressed in a space of reduced dimensions. We first found the number of components that would explain more than *p*% of the total variance in the data set for both natural activity and responses to stimulation. We then found the angle between these two vector subspaces, defined according to Bjorck and Golub (1973) (MATLAB function subspace), and used the cosine of this angle as a definition of the similarity of the subspaces. Values of similarity were recalculated for variance proportions *p* from 80 to 99%.

#### Reconstruction analysis.

Subspace similarity is an attractive measure because it is well characterized mathematically. However, it is not directly related to the issue of neuroscientific interest, which is whether outputs from the stimulated structure could generate the natural activity. To address this, we devised a novel analysis as follows.

Assume vectors of stimulus responses across different muscles are represented by ***x_i_***; where *i* indexes different stimulus sites (*i* = 1..*N_stimuli_*); these are normalized to have unit length. Vectors of natural activity patterns observed during natural behavior are represented by ***y_j_***, where *j* indexes different time points of the natural behavior (*j* = 1..*N_natural_*). Both vectors have the same dimension, which is equal to the number of EMGs used in the analysis. We then attempt to reconstruct the natural activity from the stimulus-evoked activity, according to

$$y_{j}=\overset{N_{stimuli}}{\underset{i=1}{\sum }}a_{i,j}x_{i}+\varepsilon_{j},$$

where *a_i,j_* is a set of nonnegative coefficients used to weight the stimulus evoked activities, and ***ε_j_*** is the residual, that part of ***y_j_*** which cannot be reconstructed by the vectors ***x_i_***. The coefficients were determined to minimize the residuals using linear least squares optimization with the constraint that coefficients must be nonnegative (MATLAB function lsqnonneg). We then defined two measures of performance.

The normalized length *L* was defined as

$$L=\frac{\sum_{j=1}^{N_{natural}}\sum_{i=1}^{N_{stimuli}}a_{i,j}}{\sum_{j=1}^{N_{natural}}\left|y_{j}\right|}\times 100\%.$$

If stimulus-evoked vectors line up well with natural activity vectors, then it will be possible to reach each natural activity using a direct path with a small number of stimulus vectors. The length of this path will then be no longer than the length of the vector *y_j_*, and *L* will be close to 100%. By contrast, if stimulus and natural activity vectors are poorly aligned, it will be necessary to “take the long way round” to reconstruct activities. *L* will then be substantially above 100%. *L* is a measure of how closely stimulus-evoked vectors align to the natural vectors.

Second, we defined the normalized residual *R* as

$$R=\frac{\sum_{j=1}^{N_{natural}}\left|\varepsilon_{j}\right|}{\sum_{j=1}^{N_{natural}}\left|y_{i}\right|}\times 100\text{\%}.$$

*R* measures the magnitude of the residual, i.e., that part of the natural activity that cannot be reached by the stimulus vectors, as a percentage of the total magnitude of the natural activity. If stimulus vectors can represent all patterns in the natural activity perfectly, *R* will be zero. If, by contrast, no natural activity can be represented by the stimulus vectors, then *R* will be 100%.

In comparing values of *L* and *R* across animals and areas, we faced the problem that different numbers of stimulus vectors *N_stimuli_* were available. Trivially, it will be harder to reconstruct a set of natural activities with only a small set of stimulus vectors than with a large one. We solved this by choosing a fixed number of stimulus vectors to be used and drawing this number from those available (without replacement). Values of *L* and *R* were then computed. This process was repeated 50 times, using a different subset of stimulus vectors. For a given brain or spinal cord structure, we had observations from two animals. Single values of *L* and *R* were averaged across animals, yielding sets of 50 values per brain or spinal cord area.

To determine whether *L* or *R* differed between two areas, we first computed the difference between the mean value in one area and its mean in another area. The two sets of 50 values were then shuffled and randomly assigned to areas; the difference in means between areas was then recalculated. This shuffling procedure was repeated 10,000 times. Finding what proportion of differences in means from the shuffled data were more extreme than the difference found in the actual data provided an approximate *P* value for the null hypothesis that any difference arose by chance. Values of *L* and *R* are presented as means ± SD, computed over the 50 subsets of stimulus vectors. A schematic illustration of how *L* and *R* were computed is given in the relevant part of results.

## RESULTS

**Fig. 2.**
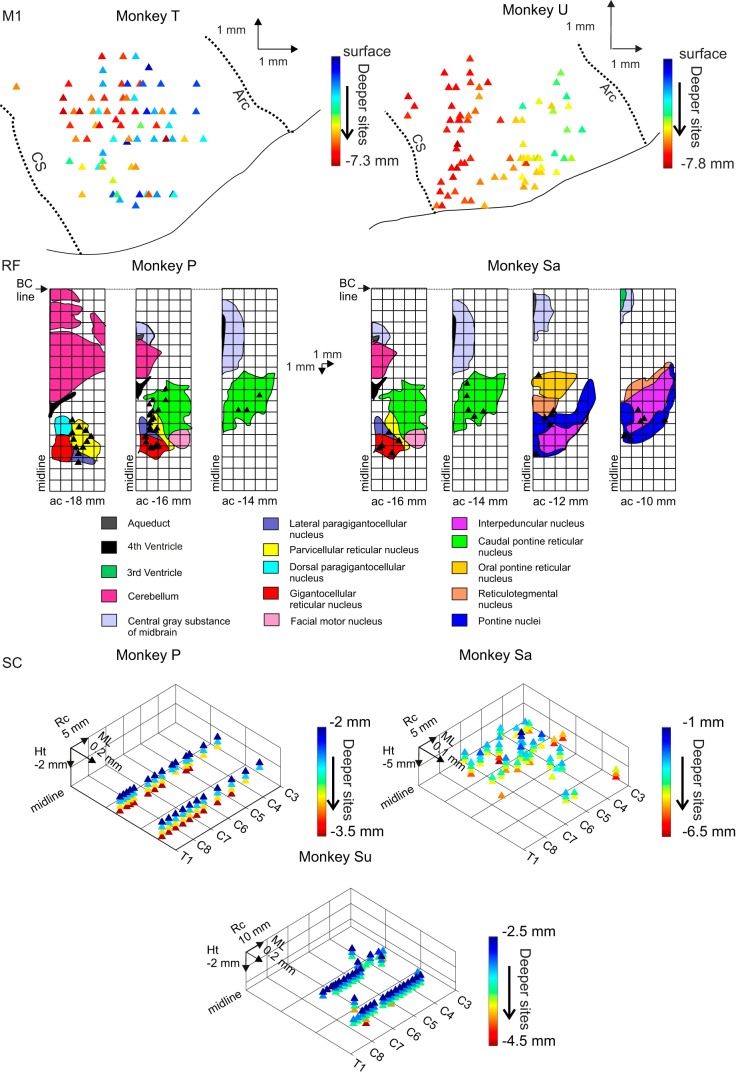
Reconstruction of stimulation sites. For M1, the calculated stereotaxic coordinates have been superimposed on tracings of cortical features based on MRI scans. For reticular formation (RF), the stereotaxic coordinates were rotated to the bicommissural (BC) plane and overlain on an atlas template with the representation of different reticular nuclei and landmarks. ac, Anterior commissure. The 3-dimensional plots show the penetrations sites in the T1-C3 segments of the spinal cord (SC). CS, central sulcus; Arc, arcuate sulcus; Rc, rostro-caudal; Ht, dorsoventral height.

Table 1 documents the recordings available for analysis in this report. In all cases, the available data comprised a range of proximal and distal muscle, acting around shoulder, elbow, wrist, and digit joints. Median stimulus intensities at threshold were 30, 30, and 59 µA, with ranges of 5–60, 5–75, and 2–200 µA for M1, RF, and SC respectively. Figure 2 shows a reconstruction of the stimulated sites.

### 

#### Divergence.

**Fig. 3.**
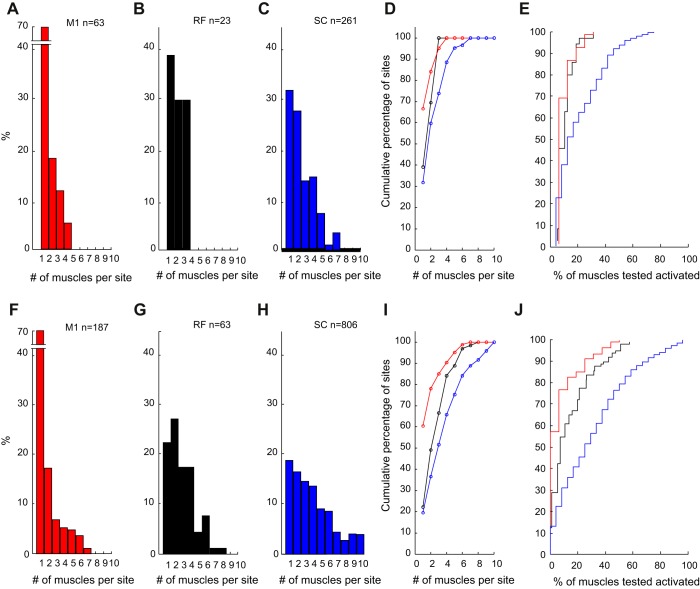
Distribution of number of muscles activated per site, using a reduced set of 10 muscles for each area, and only sites activated at threshold intensity *A*: M1. *B*: RF. *C*: SC. *D*: same data as *A*–*C*, but replotted as a cumulative distribution to allow comparison of the different areas. Line colors correspond to the colors used in *A*–*C*. *E*: similar plot as *D*, but now using all muscles recorded in a given animal; abscissa is expressed as a percentage of the number of muscles available for analysis in each case. *F*–*J*: similar display as *A*–*E*, but now using all responses recorded, rather than just those at threshold.

One of the most straightforward measures of fractionated output is to count the number of muscles that are coactivated when a given region is stimulated. We would expect that an area that subserved well-fractionated movements would be capable of activating a single muscle or perhaps a small group of synergistic muscles. Figure 3 presents the distribution of numbers of muscles activated from individual sites. One problem in comparing results between animals and areas is that different numbers of muscles were recorded in each case (see Table 1), which could bias the results.

We took two approaches to this problem. First, we selected a representative subset of 10 muscles in each animal. Figure 3, *A*–*C*, shows histograms of numbers of muscles in this subset activated by sites judged to be at threshold intensity (see methods), for M1, RF, and SC. Results have here been combined across two (M1/RF) or three animals (SC). M1 had the largest number of threshold sites that activated just one muscle (67%), but appreciable fractions of sites in RF and SC also activated single muscles (39 and 32%, respectively). The mean ± SD numbers of muscles activated were 1.5 ± 0.9, 1.9 ± 0.8, and 2.5 ± 1.6 for M1, RF, and SC respectively; these were only significantly different between M1 and SC (*P* < 10^−6^, Wilcoxon test corrected for 3 comparisons). Figure 3*D* replots these distributions as cumulative probability functions. The curves for M1 and RF, aside from their first point, are heavily overlapping. SC is distinguished by having a longer tail to its distribution, with 26% of sites activating 4 or more muscles, whereas only 4.8% of M1 and no RF sites showed such a divergent projection at threshold.

As an alternative way of compensating for different numbers of recorded muscles, Fig. 3*E* presents cumulative distributions that use all of the available muscle recordings in each animal. The abscissa for this graph shows the number of muscles activated as a percentage of the muscles recorded in that animal. In this plot, the distributions for M1 and RF are closely overlapping, with average percentage activations of 9.6 ± 6.0 and 10.7 ± 5.8%, respectively. Such a display makes the divergent nature of the spinal output even more pronounced, with activation by SC of 20.9 ± 17.0%, which differed significantly from both M1 and RF (*P* < 10^−5^ and *P* < 0.05, respectively, Wilcoxon test corrected for 3 comparisons).

Figure 3, *F*–*J*, presents data similar to Fig. 3, *A*–*E*, but now using all intensities of stimulation, and not just those judged at threshold. As expected, there is increased divergence both in the reduced subset of muscles (1.9 ± 1.5, 2.9 ± 1.7, and 3.9 ± 2.5 muscles activated for M1, RF, and SC respectively) and in the complete set (13.6 ± 12.1, 18.8 ± 14.8, and 34.5 ± 25.8% muscles activated for M1, RF, and SC respectively). All pairwise comparisons were now significantly different (*P* < 0.05, Wilcoxon test corrected for 3 comparisons).

#### Activation of different muscle groups.

**Fig. 4.**
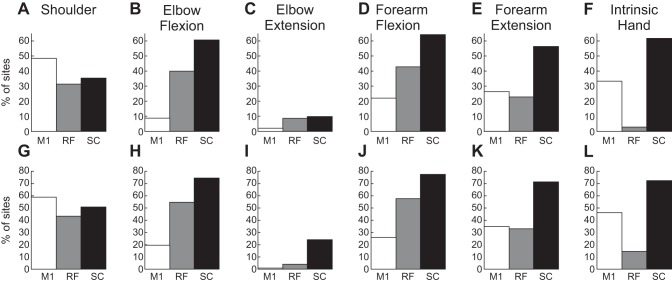
Proportions of stimulated sites that activated different muscle groups. *A*–*F*: for responses at threshold. *G*–*L*: for all responses recorded. Muscle groups are specified at the *top* of each graph.

Previous work has reported that motor outputs from different areas can be biased toward particular muscle groups. Figure 4 presents data on the proportion of sites that activated muscles classified anatomically. Results for threshold stimulus intensities are shown in Fig. 4*A*–*F*. Similar proportions of sites from M1, RF and SC activated shoulder muscles (Fig. 4*A*). At least in our data set, M1 had few projections to elbow muscles; both RF and SC activated elbow flexors more often than extensors (Fig. 4, *B* and *C*). Within the forearm, RF activated flexors more often than extensors, as previously reported (Davidson and Buford 2006), but flexion/extension differences were not seen for M1 or SC (Fig. 4*, D* and *E*). Few outputs to intrinsic hand muscles were seen from the RF, whereas M1 and SC often activated this group (Fig. 4*F*). Figure 4, *G*–*L*, presents results using all intensities of stimulation, and not just those at threshold. Although there is a general increase in the proportions of sites activating particular groups, the differences between the areas were comparable with those following threshold stimulation.

**Fig. 5.**
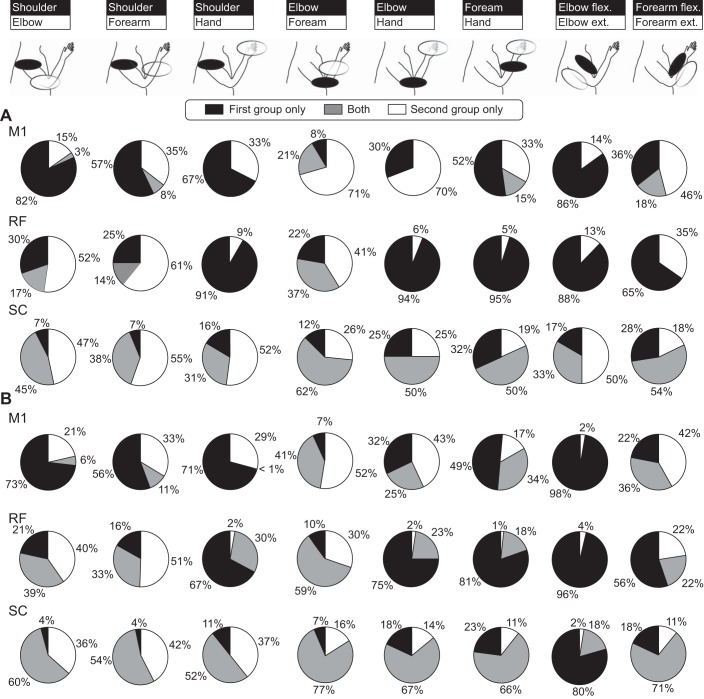
Proportions of sites that activated combinations of muscle groups singly or together. *A*: for sites stimulated at threshold. *B*: for all responses recorded. Pie charts show the results for M1, RF, and SC (rows) and different pairs of muscle groups (columns), as illustrated on the picture of an arm at the *top*.

Given the observation that many sites activated more than one muscle (Fig. 3), it is of interest to see how the coactivated muscles were organized relative to anatomic location. Figure 5 accordingly shows as pie charts the fraction of sites that activated particular groups alone or in combination. Figure 5*A* presents results for threshold stimulation; rows here indicate data from the three different areas (M1, RF, or SC), and the columns represent various pairs of muscle groups. RF only activated three pairs of muscle groups together, and then this was often a small proportion of responses. M1 also showed little propensity to activate muscles in different anatomic groups at threshold. One important difference from RF was that a small proportion (18%) of M1 sites could coactivate forearm flexors and extensors. Outputs from SC were considerably more divergent; coactivation was seen for all pairs of muscle groups examined and often formed a substantial proportion of the total output.

Figure 5*B* presents a similar analysis for all stimuli, and not just those at threshold. The increased intensity tended to increase coactivation of groups. Exceptions to this were M1 coactivation of shoulder muscles with any other group; this remained rare even at higher intensities. Also, neither M1 nor RF ever coactivated elbow flexors with extensors, even at the higher intensities.

#### Principal component analysis.

**Fig. 6.**
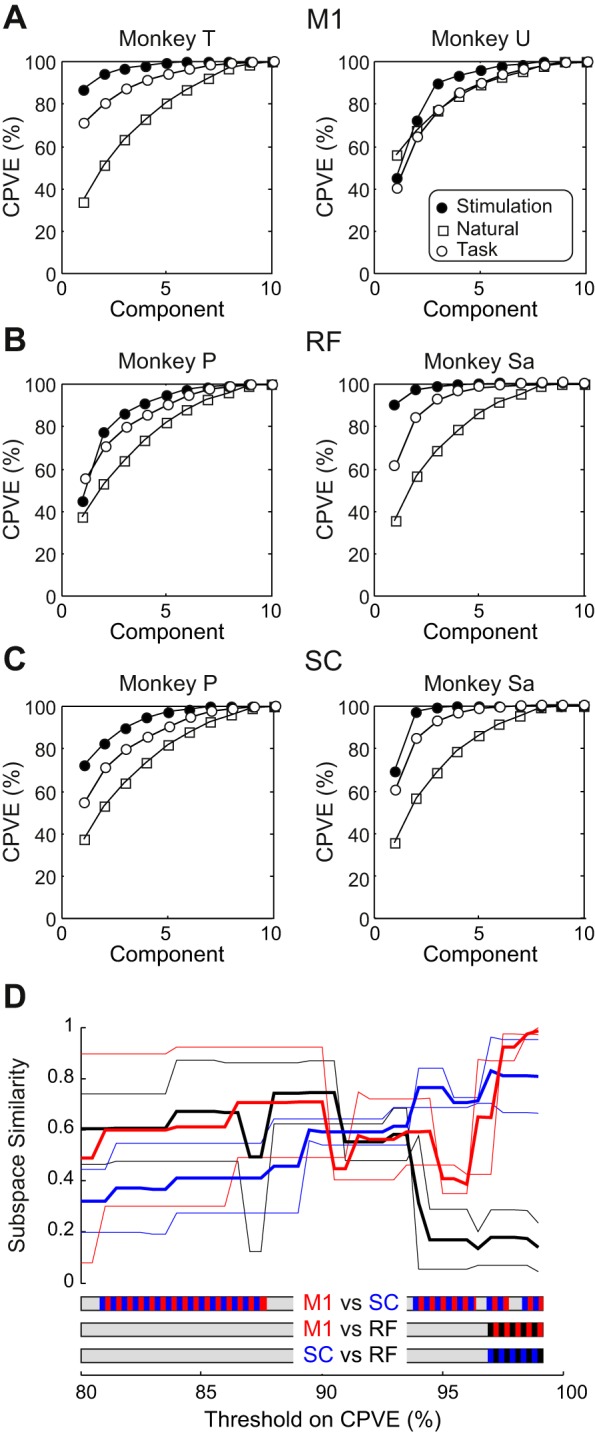
Principal component analysis of stimulus-evoked and natural activity patterns. *A*–*C*: cumulative percentage of variance explained (CPVE) as a function of the number of principal components used to reduce the dimensionality of the data. ●, Results for stimulus-evoked activity; □, results for natural activity recorded with the data logger; ○, results for activity recorded in the laboratory during performance of the reach and grasp task. Each plot shows results from a different animal and area: M1 (*A*), RF (*B*), and SC (*C*). *D*: subspace similarity. For a given CPVE value (abscissa), the number of principal components required to represent at least that CPVE was determined for both stimulus-evoked and natural activity, and the subspace similarity (see methods) was calculated between these 2 vector spaces. Thin lines show results from individual animals and areas; thick lines show results averaged across the 2 animals available for each area. Red, M1; black, RF; blue, SC. Bars at the *bottom* indicate regions where pairs of similarity measures differ significantly (*P* < 0.05).

We used principal component analysis of muscle activation patterns to explore fractionation of outputs from different areas. This was quantified by plotting the cumulative percentage of variance explained by including successive components (Dean and Baker 2017). A well fractionated output should produce a curve that rises only slowly, indicating that the muscle activation pattern cannot be well represented by a low dimensional reconstruction. Figure 6, *A*–*C*, shows such plots for each animal and area recorded. Three curves are shown on each graph, corresponding to results from the natural patterns of activity that were recorded using the data logger (□), activity patterns recorded in the laboratory during performance of the reach and grasp task (○), and stimulus-evoked patterns (●). All results used a subset of 10 muscles so that data sets had the same dimensions.

The results for natural activity patterns were broadly similar across animals. The curves for cumulative percentage of variance explained rose slowly; to explain >95% of the total variance required seven or eight components out of 10, suggesting a complex range of activations that defied compression to a small number of dimensions. The curves for activity during performance of the task in the laboratory rose more rapidly in three-quarters of the animals, which is consistent with the more constrained behavioral repertoire required during repetitive performance of an overtrained and highly stereotyped task. Between four and seven components were required to explain >95% of the variance. There was more variation for the stimulus-evoked responses; to explain >95% of the variance required two or five components for the M1, two, or four for the RF data sets, and two or four for SC. It is surprising that for each area, in one of the two animals available, >95% of variance could be explained by just two components. This suggests that, although sites often activated many muscles, these must have been quite stereotyped, so just two principal components could represent the majority of the variation. Fewer components were always needed to explain a given variance for the stimulus-evoked responses than the natural activity, suggesting that natural activity was richly fractionated.

#### Subspace similarity analysis.

Having data on both natural activity patterns and stimulus-evoked responses across multiple muscles provides an opportunity to examine how similar these were. To do this, for a given animal and area, we first found the number of principal components required to explain more than a threshold level of variance for both the stimulus evoked and natural activity patterns. We then calculated a measure of similarity between the subspaces defined by these principal components. This measure will be unity if all points in the natural activity subspace could be reached by the components that defined the stimulus-evoked subspace. By contrast, the measure will be zero if the spaces are orthogonal, meaning that they are entirely incompatible.

Figure 6*D* shows how the subspace similarity measure varied as the cutoff threshold for percentage of variance explained increased. The thin lines show results for different animals, with the line color indicating the area (red, M1; black, RF; blue, SC); the thick lines show the average across the two animals available for each area. Below 95% of variance explained, the lines were intermixed, and there appeared to be no clear separation between the different areas. However, above 95%, the similarity for both RF data sets reduced to low values and remained below the similarity for M1 and SC. Differences between RF-M1 and RF-SC were significant above 96.5% of variance explained (see bars beneath the plots, *P* < 0.05; Fig. 6*D*). This suggests that RF is poorly able to support the full extent of variation seen in natural activity compared with M1 and SC. Significant differences between SC and M1 were seen over a wider range of thresholds.

#### Reconstruction of natural activity from stimulus-evoked activity.

**Fig. 7.**
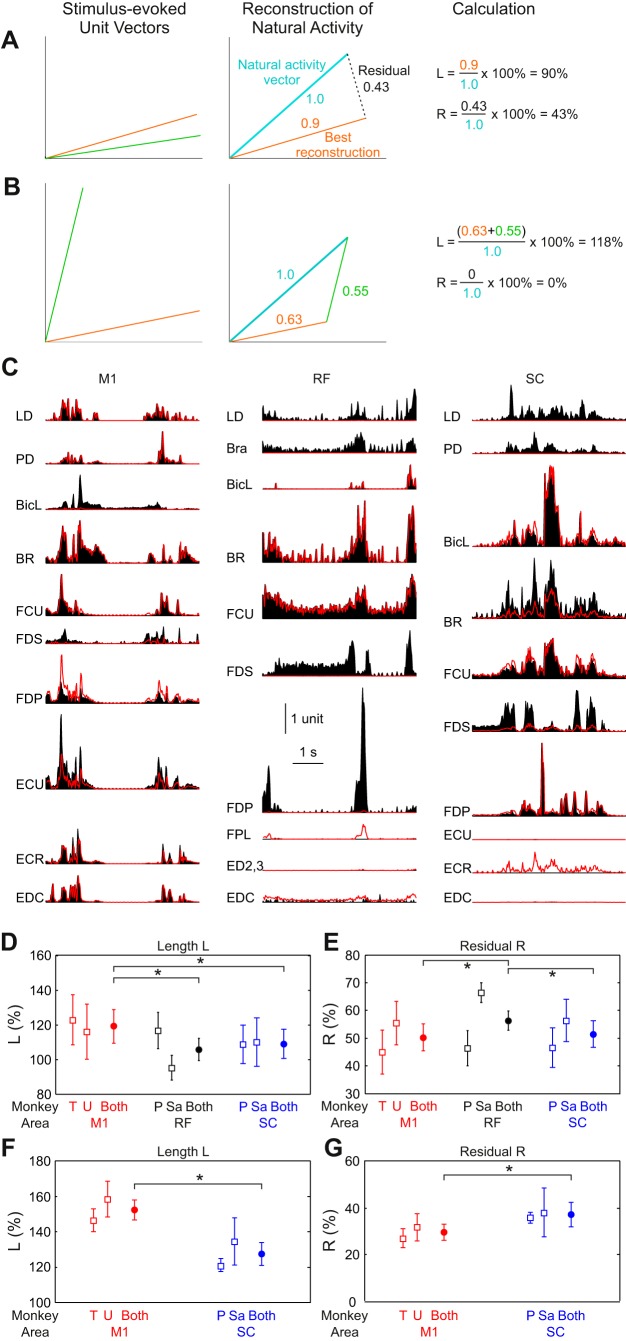
Reconstruction of natural activity from stimulus-evoked activity. *A* and *B*: schematic illustration of the analysis approach. Unit vectors representing stimulus-evoked activity (*left panel*, green, orange) are combined to reconstruct natural activity (*middle panel*, blue). The total length of the reconstruction as a percentage of the length of the natural activity provides the normalized length measure *L*. The size of the residual as a percentage of the length of the natural activity provides the normalized residual measure *R*. For display purposes, 2-dimensional vectors are shown; in reality, the vectors are in 10 dimensions, corresponding to the activity of 10 muscles. In *A*, a situation where natural activity cannot be reconstructed perfectly is shown, so a residual (error) term remains. In *B*, perfect reconstruction is possible. *C*: example reconstructions of short sections of natural EMG activity from M1 (*monkey T*), RF (*monkey Sa*), and SC (*monkey P*). Black traces show the recorded data; red traces show the reconstructions from stimulus-evoked vectors; 1 unit in the vertical calibration bar corresponds to the EMG 99th percentile. *D*: values of *L* calculated from this analysis using subsets of 30 stimulus-evoked responses. *E*: corresponding values of *R*. *F*: values of *L* calculated using subsets of 80 stimulus-evoked responses. *G*: corresponding values of *R*. □, Results from individual animals and areas; ●, average across 2 animals available for each area. Error bars indicate the standard deviation, computed with 50 different randomly drawn subsets of stimulus-evoked vectors. Brackets indicate significant pairwise differences between areas (*P* < 10^−4^; Monte Carlo test).

One problem with the principal component and subspace analysis is that it does not take account of the fixed sign of the output effects. If one stimulus site coactivates two muscles, it is not the case that it can also suppress them together as well. To address this limitation, we also designed an analysis that sought to reconstruct the natural activity patterns, using only positively scaled combinations of the stimulus-evoked activities. This is described in methods and illustrated in Fig. 7, *A* and *B*.

We began with the set of stimulus-evoked muscle response patterns, which were represented as vectors in the 10-dimensional muscle space. In the schematic representation of Fig. 7, *A* and *B*, only two stimulus-evoked vectors are shown in two dimensions; these are normalized to have unit length. At any given time point during recordings with the data logger in the home cage, the natural muscle activity will also form a vector (Fig. 7, *A* and *B*, blue line, *middle*). We attempt to reconstruct this using a sum of stimulus vectors scaled by positive weights. For the example in Fig. 7*A*, an exact reconstruction is not possible; the residual is the difference between the closest point that can be reached using the stimulus vectors and the natural activity (Fig. 7*A*, *middle*, dotted black line). For the example in Fig. 7*B*, the stimulus vectors do allow an exact reconstruction.

As described in methods, we computed two quantities; example computation of these is shown in Fig. 7, *A* and *B*, right. The normalized vector length (*L*) represented how efficiently the natural activity can be generated. *L* will be close to 100% if the stimulus activity vectors point in the same direction as the natural activity vectors but exceed 100% if there are substantial differences, requiring reconstruction to “take the long way round” (see Fig. 7*B*). The normalized residual (*R*) indicates what proportion of the natural activity was unreachable from the stimulus-evoked activity.

A larger number of stimulus evoked activities would permit better reconstruction of the natural activity; this could lead to artifactual results, as recordings from different areas had different numbers of responses. We corrected for this by drawing a fixed number of responses from those available for all areas; this was repeated 50 times, and the results averaged across the two animals in each area had been recorded.

Figure 7*C* shows examples of natural activity patterns (black) and the reconstructions generated from stimulus-evoked vectors from M1, RF, and SC when using a subset of 30 stimulus vectors. To produce this display, we chose the stimulus vector subset out of the 50 repetitions that gave values of *L* and *R* closest to the mean in that animal. The 5-s-long time window shown has also been chosen to have computed *L* and *R* values close to the mean over the entire recording. We can distinguish two types of error in the reconstruction. First, activity in a given muscle may not be present in the reconstruction at all, for example, the LD, Bra, and FDS muscles for the RF reconstruction. Second, in some instances, muscles are activated in the reconstruction more than required, such as the bottom muscle EDC for RF and ECR for SC. Both errors reflect limitations in the ability of the stimulus vectors to reach all parts of the natural activity space. Minimizing reconstruction error then requires a tradeoff, as the only way to produce required activity is to generate unwanted cocontractions. In these illustrations, the M1 reconstruction fitted the natural activity closer than RF and SC.

Figure 7*D* shows the results for normalized length *L*, and Fig. 7*E* shows the results for normalized residual *R*, when a subset of 30 responses was used. The normalized length *L* was 119.1 ± 9.6, 105.9 ± 6.2, and 109.3 ± 8.4% for M1, RF, and SC respectively (means ± SD of repetitions). The length for M1 was significantly different from the other two areas (*P* < 10^−4^; Monte Carlo test); differences between the lengths for RF and SC just failed to reach significance (*P* = 0.072; Monte Carlo test corrected for 3 pairwise comparisons). The normalized residual *R* was 50.3 ± 4.9, 56.4 ± 3.5, and 51.5 ± 4.8% for M1, RF, and SC, respectively (means ± SD). The residual for RF was significantly different from that for the other two areas (*P* < 10^−4^; Monte Carlo test).

The choice of a relatively small number of stimulus responses (30) for the above analysis was dictated by the small number available for the RF (see Table 1). Therefore, the analysis was repeated using sets of 80 stimulus responses, but now including only M1 and SC responses; the results of this are shown in Fig. 7, *F* and *G*. The normalized length *L* was 152.4 ± 5.7% for M1 and 127.7 ± 6.5% for SC. The normalized residual *R* was 29.6 ± 3.5% for M1 and 37.3 ± 5.3% for SC. M1 and SC differed significantly on both measures (*P* < 10^−4^; Monte Carlo test).

It is clear that the detailed numbers produced by this analysis are highly dependent on the choice of the number of responses to use. However, a consistent finding was that M1 had higher lengths *L* than RF and SC. The residuals *R* were ordered, with M1 being lowest, RF being highest, and SC intermediate between the two.

## DISCUSSION

### 

#### Measures of output divergence.

Previous work has examined the outputs from different motor structures using responses to either stimulation or post-spike facilitation in spike-triggered averages of rectified EMG. Often similar proportions of muscles were reported to be activated from different motor structures, although the interpretation of these numbers varied widely. Thus Buys et al. (1986) reported that a single cortico-motoneuronal cell in M1 facilitated on average 27% of the muscles recorded and concluded that “cortico-motor neurones may contribute to relatively independent finger movements by virtue of their selective facilitation of hand muscles, leading to a fractionated pattern of muscle activity.” Takei and Seki (2010) found that an average of 20% of muscles were facilitated by a single spinal cell (rising to 45% when only intrinsic hand muscles were considered) and concluded that “these divergent facilitations … could help form ‘functional units’ for hand movements.” That similar proportions should have led to opposite interpretations perhaps emphasizes the importance of a direct comparison of different areas, as in the present work.

In this study, we used weak single pulse stimuli to estimate output effects. This provided a greater sample of possible output combinations than would have been possible by examining post-spike facilitations, where the yield of cells with monosynaptic connections to motoneuron pools can be low. In the cortex, we know that weak ICMS activates a small population of output neurons, because post-stimulus facilitations are larger than post-spike facilitations (Cheney and Fetz 1985). The activated population may spread over several millimeters of cortical surface (Baker et al. 1998; Hao et al. 2016). Because we have not assessed output divergence at the finest-grain possible (the single cell), this may have increased the number of coactivated muscles observed. However, in cortex, stimuli appear to activate functionally similar cortico-motoneuronal cells (Cheney and Fetz 1985), presumably reflecting that functionally related cells share common inputs (Jackson et al. 2003). Therefore, weak stimuli may be accessing functional output modules in a manner not dissimilar from natural activation. Examining post-spike facilitations is itself not without problems. Nonlinearities in the rectified EMG make weak effects especially hard to detect (Baker and Lemon 1995, 1998); therefore, examining post-spike facilitation from single cells is likely to underestimate the output divergence.

Clearly as stimulus intensity is increased, the number of activated output neurons will rise. A previous study (Park et al. 2004) used a fixed intensity of 15 μA as a reasonable compromise between obtaining clear and consistent effects while limiting the loss of specificity due to stimulus spread. In this work, we adjusted the intensity to be just above threshold, yielding the smallest possible spread of the effects. Naturally this approach could also suffer from a failure to detect weak effects, and (as for spike triggered averaging) underestimate divergence. Nevertheless, the results of Fig. 3, *A*–*E*, probably provide the best comparison so far available of the size of muscles groups activated by M1, RF, and SC. Surprisingly, the number of muscles activated by M1 and RF following threshold stimuli was comparable, but both brain areas activated fewer muscles on average than sites in the spinal cord.

Recordings from M1 and RF were carried out in the conscious state, whereas the data from SC were gathered under anesthesia. We might expect that anesthesia would suppress spinal interneuron and motoneuron circuits, and indeed, there was little or no background EMG in the anesthetized recordings, whereas responses in the conscious state during task performance had active muscles. This could have reduced the number of muscles affected, possibly making outputs appear more stereotyped. However, on average, SC activated more muscles than either of the other two centers (Fig. 3), and reconstruction of natural activity was midway between RF and M1 (Fig. 7). Although we cannot rule out an effect of anesthesia on the detailed numbers that we report, it seems unlikely that our qualitative conclusions have been materially affected.

More subtle changes in the excitability of the activated circuits were also likely to occur at different phases of task performance, when muscles went from inactive to active at different levels and in combination with different synergists (Fig. 1*E*). The efficacy of monosynaptic cortico-motoneuronal outputs can modulate with different task phases (Davidson et al. 2007a); outputs with less direct linkage are even more likely to change their efficacy with task (Dyson et al. 2014). In common with previous work, we simply averaged all stimuli occurring at different task phases. This is likely to give the best estimate of the potential output from one site. Nevertheless, it remains possible that highly transient effects were diluted by averaging with periods where there was no effect, which could have reduced them so that they fell below the detection threshold.

#### Flexible encoding vs. fixed synergies.

Previous work on neural synergies has used approaches like principal component analysis to test whether a high-dimensional pattern of muscle activities can be expressed in a smaller number of dimensions. Used alone, we did not find obvious differences in the results of principal component analysis between areas (Fig. 6, *A*–*C*, ●). This may reflect the arbitrary sampling of sites in our experiment. The directions of maximal variance in the stimulus-evoked activity vectors may reflect this random selection. For example, if several sites by chance generate similar effects, this would allow a high proportion of variance across the data set to be represented by a small number of components. Yet the variation seen in the undersampled sites might be of critical importance to the function of that area. Relevant to this, Santello et al. (1998) found that ~85% of the variance in their data set of hand postures could be represented by just two principal components. However, although the higher components contributed negligible fractions to the variance, they were of critical importance in coding the difference between hand postures. For this reason, we performed two analyses that compared stimulus-evoked muscle activity patterns with those occurring during unconstrained behavior in the home cage.

When data sets were reduced in dimensionality to represent ≤90% of the variance, the similarity between muscle-evoked and naturally occurring activity showed no consistent difference between areas (Fig. 6*D*). However, when we considered subspaces that represented >95% of the variance, there was a clear separation, with the patterns evoked by RF stimulation showing lower similarity to natural activity compared with M1 and SC.

Second, we developed a novel analysis that tested how well natural patterns could be generated from stimulus-evoked patterns. The results seemed to show a hierarchy. RF sites could efficiently generate an approximation to natural activity; the normalized vector lengths *L* were lower than for M1 and SC (Fig. 7*D*), meaning that activation of an RF site could take activity more directly toward the desired point. However, the normalized residuals *R* were larger for RF than for M1 and SC (Fig. 7*E*); relying on RF alone would result in more errors, producing only an approximation to the desired activity pattern. This suggests the same conclusion as the subspace similarity analysis; accurate generation of natural activity is poorly served by RF alone. Comparison of M1 and SC using the larger data sets available for these areas showed some differences, with M1 producing less efficient (high *L*) but more accurate (low *R*) reconstruction of natural activity than SC, although these differences were less marked than between RF and M1/SC.

Although we found significant differences between areas using these measures, differences were small (e.g., *L* = 119.1 vs. 105.9% for M1 vs. RF). This may reflect that all areas are reasonably matched to the gross structure (i.e., majority of the variance) of natural movements. However, the small differences may also reflect our limited sampling of the outputs of each region. When we used subsets of 80 stimulus-evoked vectors, the differences between M1 and SC became larger (*L* = 152.4 vs. 127.7% compared with *L* = 119.1 vs. 109.3% when only 30 vectors were used). We speculate that if we were able to sample all of the outputs, reconstruction errors from M1 would become very small, leading to negligible *R* values, whereas even with complete sampling RF would be unable to produce some natural activities accurately.

These results may agree with the changes in upper limb function that are seen following a corticospinal tract lesion either following experimental pyramidal tract section in monkey (Alstermark et al. 2011; Lawrence and Kuypers 1968) or after stroke in human patients (Lang and Schieber 2003). After an initial flaccid paralysis, strength recovers partly by the strengthening of reticulospinal pathways (Zaaimi et al. 2012). However, there is a permanent deficit in independent finger movements and unhelpful obligate coupling between muscle groups to form detrimental synergies (Dewald et al. 1995). Our data support previous suggestions that these deficits represent the less precise activation of specific muscle patterns by surviving subcortical pathways compared with the corticospinal tract (Baker et al. 2015; Dewald et al. 1995; Lawrence and Kuypers 1968). Interestingly, similar muscle activation synergies can be extracted from natural movements after stroke on the affected and unaffected side (Cheung et al. 2009). This is to be expected if these synergies reflect the first-pass approximations to desired movements achieved by reticulospinal outputs; the affected side has an impaired corticospinal contribution, which may add little to the overall variance but is critical for accurate movement performance. Recent work by Xu et al. (2017) proposed that two separate systems drive recovery post-stroke; one is responsible for all of strength recovery and some fractionation, and the other generates additional recovery in fractionation. Our work supports the tentative assignment of the first system to RF and the second to M1 outputs.

Observations after recovery from lesions suggest that spinal circuits occupy a position intermediate between cortical and brainstem centers. After a corticospinal lesion at segmental level C5, monkeys can recover precision grip (Sasaki et al. 2004); this is not possible after lesions at C2 (Alstermark et al. 2011). The difference is likely to be the spared inputs to propriospinal neurons at C3–C4 in the former case, demonstrating that these cells can mediate some fine finger movements. However, these animals alter their muscle activation patterns after the lesion, showing more cocontraction of antagonists (Nishimura et al. 2009), suggesting that there is still some residual deficit in fractionation compared with the intact system with fully functioning corticospinal tract.

This study did not consider stimulus-evoked suppression of muscle activity for practical reasons. Inhibition is typically harder to detect statistically in correlation or average measures (Aertsen and Gerstein 1985). Because we sought effects just above threshold, significant suppression was only rarely seen. In the spinal experiments under anesthesia, no suppressions could be evaluated due to the lack of background activity. Rather than include the arbitrary small number of effects detected, we concentrated solely on stimulus-evoked facilitation. However, suppression of unwanted muscles is likely to be an important means of focusing activation and thereby achieving fractionation. This is known to occur at the cortical level, where antagonizing intracortical GABAergic transmission results in an expansion of the output map (Jacobs and Donoghue 1991). Both M1 and RF generate stimulus-evoked suppression of muscle activity (Cheney et al. 1985; Davidson and Buford 2004) via effects on inhibitory interneurons in the SC (Jankowska and Tanaka 1974; Jankowska et al. 1968). We do not have any information on the relative contributions to fractionation of inhibition from the different motor centers.

This work has considered outputs from M1, RF, and SC independently. However, we know that these areas are connected in a hierarchy; M1 provides corticoreticular input to RF (Keizer and Kuypers 1989) and corticospinal input to SC interneurons (Yanai et al. 2007), which also receive input from RF (Riddle and Baker 2010). It is likely that the weak cortical stimuli that we used generate effects mainly via the monosynaptic cortico-motoneuronal connections. However, during natural activity, outputs will be generated by the multiplicity of connections between M1 and motoneurons, including the RF and SC circuits, which we have examined here. In addition, premotor cortical areas such as SMA and F5 also project to the RF (Keizer and Kuypers 1989) and to SC interneurons (Borra et al. 2010; McNeal et al. 2010), providing a further route by which subcortical systems can be controlled independently of M1. The coordinated action of these many pathways lead together to fractionated movements in a healthy primate.

## GRANTS

This work was supported by Medical Research Council Grant No. MR/J012688/1, and Wellcome Trust Grant No. 101002.

## DISCLOSURES

No conflicts of interest, financial or otherwise, are declared by the authors.

## AUTHOR CONTRIBUTIONS

B.Z., L.R.D., and S.N.B. performed experiments; B.Z., L.R.D., and S.N.B. analyzed data; B.Z., L.R.D., and S.N.B. interpreted results of experiments; B.Z., L.R.D., and S.N.B. prepared figures; B.Z., L.R.D., and S.N.B. edited and revised manuscript; B.Z., L.R.D., and S.N.B. approved final version of manuscript; S.N.B. conceived and designed research; S.N.B. drafted manuscript.

## References

[B1] Aertsen AM, Gerstein GL Evaluation of neuronal connectivity: sensitivity of cross-correlation. Brain Res 340: 341–354, 1985. doi:10.1016/0006-8993(85)90931-X. 4027655

[B2] Alstermark B, Pettersson LG, Nishimura Y, Yoshino-Saito K, Tsuboi F, Takahashi M, Isa T Motor command for precision grip in the macaque monkey can be mediated by spinal interneurons. J Neurophysiol 106: 122–126, 2011. doi:10.1152/jn.00089.2011. 21511706

[B3] Armand J, Olivier E, Edgley SA, Lemon RN Postnatal development of corticospinal projections from motor cortex to the cervical enlargement in the macaque monkey. J Neurosci 17: 251–266, 1997. 8987753 10.1523/JNEUROSCI.17-01-00251.1997 PMC6793701

[B4] Baker SN, Lemon RN Non-linear summation of responses in averages of rectified EMG. J Neurosci Methods 59: 175–181, 1995. doi:10.1016/0165-0270(94)00180-O. 8531484

[B5] Baker SN, Lemon RN Computer simulation of post-spike facilitation in spike-triggered averages of rectified EMG. J Neurophysiol 80: 1391–1406, 1998. 9744948 10.1152/jn.1998.80.3.1391

[B6] Baker SN, Olivier E, Lemon RN An investigation of the intrinsic circuitry of the motor cortex of the monkey using intra-cortical microstimulation. Exp Brain Res 123: 397–411, 1998. doi:10.1007/s002210050585. 9870600

[B7] Baker SN, Philbin N, Spinks R, Pinches EM, Wolpert DM, MacManus DG, Pauluis Q, Lemon RN Multiple single unit recording in the cortex of monkeys using independently moveable microelectrodes. J Neurosci Methods 94: 5–17, 1999. doi:10.1016/S0165-0270(99)00121-1. 10638811

[B8] Baker SN, Zaaimi B, Fisher KM, Edgley SA, Soteropoulos DS Pathways mediating functional recovery. Prog Brain Res 218: 389–412, 2015. doi:10.1016/bs.pbr.2014.12.010. 25890147

[B9] Belhaj-Saïf A, Karrer JH, Cheney PD Distribution and characteristics of poststimulus effects in proximal and distal forelimb muscles from red nucleus in the monkey. J Neurophysiol 79: 1777–1789, 1998. 9535947 10.1152/jn.1998.79.4.1777

[B10] Bjorck A, Golub GH Numerical methods for computing angles between linear subspaces. Math Comput 27: 579–594, 1973. doi:10.2307/2005662.

[B11] Borra E, Belmalih A, Gerbella M, Rozzi S, Luppino G Projections of the hand field of the macaque ventral premotor area F5 to the brainstem and spinal cord. J Comp Neurol 518: 2570–2591, 2010. doi:10.1002/cne.22353. 20503428

[B12] Bortoff GA, Strick PL Corticospinal terminations in two new-world primates: further evidence that corticomotoneuronal connections provide part of the neural substrate for manual dexterity. J Neurosci 13: 5105–5118, 1993. 7504721 10.1523/JNEUROSCI.13-12-05105.1993 PMC6576412

[B13] Boudrias MH, Lee SP, Svojanovsky S, Cheney PD Forelimb muscle representations and output properties of motor areas in the mesial wall of rhesus macaques. Cereb Cortex 20: 704–719, 2010 a. doi:10.1093/cercor/bhp136. 19633176 PMC2820706

[B14] Boudrias MH, McPherson RL, Frost SB, Cheney PD Output properties and organization of the forelimb representation of motor areas on the lateral aspect of the hemisphere in rhesus macaques. Cereb Cortex 20: 169–186, 2010 b. doi:10.1093/cercor/bhp084. 19561063 PMC2792191

[B15] Buys EJ, Lemon RN, Mantel GWH, Muir RB Selective facilitation of different hand muscles by single corticospinal neurones in the conscious monkey. J Physiol 381: 529–549, 1986. doi:10.1113/jphysiol.1986.sp016342. 3625544 PMC1182994

[B16] Cheney PD, Fetz EE Functional classes of primate corticomotoneuronal cells and their relation to active force. J Neurophysiol 44: 773–791, 1980. 6253605 10.1152/jn.1980.44.4.773

[B17] Cheney PD, Fetz EE Comparable patterns of muscle facilitation evoked by individual corticomotoneuronal (CM) cells and by single intracortical microstimuli in primates: evidence for functional groups of CM cells. J Neurophysiol 53: 786–804, 1985. 2984354 10.1152/jn.1985.53.3.786

[B18] Cheney PD, Fetz EE, Palmer SS Patterns of facilitation and suppression of antagonist forelimb muscles from motor cortex sites in the awake monkey. J Neurophysiol 53: 805–820, 1985. 2984355 10.1152/jn.1985.53.3.805

[B19] Cheung VC, Piron L, Agostini M, Silvoni S, Turolla A, Bizzi E Stability of muscle synergies for voluntary actions after cortical stroke in humans. Proc Natl Acad Sci USA 106: 19563–19568, 2009. doi:10.1073/pnas.0910114106. 19880747 PMC2780765

[B20] Davidson AG, Buford JA Motor outputs from the primate reticular formation to shoulder muscles as revealed by stimulus-triggered averaging. J Neurophysiol 92: 83–95, 2004. doi:10.1152/jn.00083.2003. 15014106 PMC2740726

[B21] Davidson AG, Buford JA Bilateral actions of the reticulospinal tract on arm and shoulder muscles in the monkey: stimulus triggered averaging. Exp Brain Res 173: 25–39, 2006. doi:10.1007/s00221-006-0374-1. 16506008

[B22] Davidson AG, Chan V, O’Dell R, Schieber MH Rapid changes in throughput from single motor cortex neurons to muscle activity. Science 318: 1934–1937, 2007 a. doi:10.1126/science.1149774. 18096808

[B23] Davidson AG, Schieber MH, Buford JA Bilateral spike-triggered average effects in arm and shoulder muscles from the monkey pontomedullary reticular formation. J Neurosci 27: 8053–8058, 2007 b. doi:10.1523/JNEUROSCI.0040-07.2007. 17652596 PMC6672715

[B24] Dean LR, Baker SN Fractionation of muscle activity in rapid responses to startling cues. J Neurophysiol 117: 1713–1719, 2017. doi:10.1152/jn.01009.2015. 28003416 PMC5384977

[B25] Dewald JP, Pope PS, Given JD, Buchanan TS, Rymer WZ Abnormal muscle coactivation patterns during isometric torque generation at the elbow and shoulder in hemiparetic subjects. Brain 118: 495–510, 1995. doi:10.1093/brain/118.2.495. 7735890

[B26] Dyson KS, Miron JP, Drew T Differential modulation of descending signals from the reticulospinal system during reaching and locomotion. J Neurophysiol 112: 2505–2528, 2014. doi:10.1152/jn.00188.2014. 25143539

[B27] Hao Y, Riehle A, Brochier TG Mapping horizontal spread of activity in monkey motor cortex using single pulse microstimulation. Front Neural Circuits 10: 104, 2016. doi:10.3389/fncir.2016.00104. 28018182 PMC5159418

[B28] Illert M, Jankowska E, Lundberg A, Odutola A Integration in descending motor pathways controlling the forelimb in the cat. 7. Effects from the reticular formation on C3–C4 propriospinal neurones. Exp Brain Res 42: 269–281, 1981. 7238671 10.1007/BF00237494

[B29] Ingram JN, Körding KP, Howard IS, Wolpert DM The statistics of natural hand movements. Exp Brain Res 188: 223–236, 2008. doi:10.1007/s00221-008-1355-3. 18369608 PMC2636901

[B30] Isa T, Ohki Y, Seki K, Alstermark B Properties of propriospinal neurons in the C3–C4 segments mediating disynaptic pyramidal excitation to forelimb motoneurons in the macaque monkey. J Neurophysiol 95: 3674–3685, 2006. doi:10.1152/jn.00103.2005. 16495365

[B31] Jackson A, Gee VJ, Baker SN, Lemon RN Synchrony between neurons with similar muscle fields in monkey motor cortex. Neuron 38: 115–125, 2003. doi:10.1016/S0896-6273(03)00162-4. 12691669

[B32] Jacobs KM, Donoghue JP Reshaping the cortical motor map by unmasking latent intracortical connections. Science 251: 944–947, 1991. doi:10.1126/science.2000496. 2000496

[B33] Jankowska E, Lund S, Lundberg A, Pompeiano O Inhibitory effects evoked through ventral reticulospinal pathways. Arch Ital Biol 106: 124–140, 1968. 4300743

[B34] Jankowska E, Tanaka R Neuronal mechanism of the disynaptic inhibition evoked in primate spinal motoneurones from the corticospinal tract. Brain Res 75: 163–166, 1974. doi:10.1016/0006-8993(74)90778-1. 4210316

[B35] Kamper DG, Rymer WZ Quantitative features of the stretch response of extrinsic finger muscles in hemiparetic stroke. Muscle Nerve 23: 954–961, 2000. doi:10.1002/(SICI)1097-4598(200006)23:6<954::AID-MUS17>3.0.CO;2-0. 10842274

[B36] Keizer K, Kuypers HGJM Distribution of corticospinal neurons with collaterals to the lower brain stem reticular formation in monkey (Macaca fascicularis). Exp Brain Res 74: 311–318, 1989. doi:10.1007/BF00248864. 2924851

[B37] Kinoshita M, Matsui R, Kato S, Hasegawa T, Kasahara H, Isa K, Watakabe A, Yamamori T, Nishimura Y, Alstermark B, Watanabe D, Kobayashi K, Isa T Genetic dissection of the circuit for hand dexterity in primates. Nature 487: 235–238, 2012. doi:10.1038/nature11206. 22722837

[B38] Kirsch E, Rivlis G, Schieber MH Primary motor cortex neurons during individuated finger and wrist movements: correlation of spike firing rates with the motion of individual digits versus their principal components. Front Neurol 5: 70, 2014. doi:10.3389/fneur.2014.00070. 24904516 PMC4032981

[B39] Kutch JJ, Kuo AD, Bloch AM, Rymer WZ Endpoint force fluctuations reveal flexible rather than synergistic patterns of muscle cooperation. J Neurophysiol 100: 2455–2471, 2008. doi:10.1152/jn.90274.2008. 18799603 PMC2585402

[B40] Kutch JJ, Valero-Cuevas FJ Challenges and new approaches to proving the existence of muscle synergies of neural origin. PLOS Comput Biol 8: e1002434, 2012. doi:10.1371/journal.pcbi.1002434. 22570602 PMC3342930

[B41] Lang CE, Schieber MH Differential impairment of individuated finger movements in humans after damage to the motor cortex or the corticospinal tract. J Neurophysiol 90: 1160–1170, 2003. doi:10.1152/jn.00130.2003. 14668295

[B42] Lawrence DG, Kuypers HGJM The functional organization of the motor system in the monkey. I. The effects of bilateral pyramidal lesions. Brain 91: 1–14, 1968. doi:10.1093/brain/91.1.1. 4966862

[B43] Lemon RN Methods for Neuronal Recording in Conscious Animals. London: Wiley, 1984.

[B44] Maier MA, Olivier E, Baker SN, Kirkwood PA, Morris T, Lemon RN Direct and indirect corticospinal control of arm and hand motoneurons in the squirrel monkey (Saimiri sciureus). J Neurophysiol 78: 721–733, 1997. 9307107 10.1152/jn.1997.78.2.721

[B45] Martin RF, Bowden DM A stereotaxic template atlas of the macaque brain for digital imaging and quantitative neuroanatomy. Neuroimage 4: 119–150, 1996. doi:10.1006/nimg.1996.0036. 9345504

[B46] McNeal DW, Darling WG, Ge J, Stilwell-Morecraft KS, Solon KM, Hynes SM, Pizzimenti MA, Rotella DL, Vanadurongvan T, Morecraft RJ Selective long-term reorganization of the corticospinal projection from the supplementary motor cortex following recovery from lateral motor cortex injury. J Comp Neurol 518: 586–621, 2010. doi:10.1002/cne.22218. 20034062 PMC3765018

[B47] Mewes K, Cheney PD Facilitation and suppression of wrist and digit muscles from single rubromotoneuronal cells in the awake monkey. J Neurophysiol 66: 1965–1977, 1991. 1812229 10.1152/jn.1991.66.6.1965

[B48] Moritz CT, Lucas TH, Perlmutter SI, Fetz EE Forelimb movements and muscle responses evoked by microstimulation of cervical spinal cord in sedated monkeys. J Neurophysiol 97: 110–120, 2007. doi:10.1152/jn.00414.2006. 16971685

[B49] Muir RB, Lemon RN Corticospinal neurons with a special role in precision grip. Brain Res 261: 312–316, 1983. doi:10.1016/0006-8993(83)90635-2. 6831213

[B50] Nishimura Y, Morichika Y, Isa T A subcortical oscillatory network contributes to recovery of hand dexterity after spinal cord injury. Brain 132: 709–721, 2009. doi:10.1093/brain/awn338. 19155271 PMC2664448

[B51] Overduin SA, d’Avella A, Carmena JM, Bizzi E Microstimulation activates a handful of muscle synergies. Neuron 76: 1071–1077, 2012. doi:10.1016/j.neuron.2012.10.018. 23259944 PMC3547640

[B52] Overduin SA, d’Avella A, Roh J, Carmena JM, Bizzi E Representation of muscle synergies in the primate brain. J Neurosci 35: 12615–12624, 2015. doi:10.1523/JNEUROSCI.4302-14.2015. 26377453 PMC4571600

[B53] Park MC, Belhaj-Saïf A, Cheney PD Properties of primary motor cortex output to forelimb muscles in rhesus macaques. J Neurophysiol 92: 2968–2984, 2004. doi:10.1152/jn.00649.2003. 15163675

[B54] Peterson BW, Maunz RA, Pitts NG, Mackel RG Patterns of projection and braching of reticulospinal neurons. Exp Brain Res 23: 333–351, 1975. doi:10.1007/BF00238019. 1183508

[B55] Raghavan P, Petra E, Krakauer JW, Gordon AM Patterns of impairment in digit independence after subcortical stroke. J Neurophysiol 95: 369–378, 2006. doi:10.1152/jn.00873.2005. 16207778

[B56] Riddle CN, Baker SN Convergence of pyramidal and medial brain stem descending pathways onto macaque cervical spinal interneurons. J Neurophysiol 103: 2821–2832, 2010. doi:10.1152/jn.00491.2009. 20457863 PMC2867561

[B57] Riddle CN, Edgley SA, Baker SN Direct and indirect connections with upper limb motoneurons from the primate reticulospinal tract. J Neurosci 29: 4993–4999, 2009. doi:10.1523/JNEUROSCI.3720-08.2009. 19369568 PMC2690979

[B58] Roh J, Rymer WZ, Perreault EJ, Yoo SB, Beer RF Alterations in upper limb muscle synergy structure in chronic stroke survivors. J Neurophysiol 109: 768–781, 2013. doi:10.1152/jn.00670.2012. 23155178 PMC3567389

[B59] Saltiel P, Wyler-Duda K, D’Avella A, Tresch MC, Bizzi E Muscle synergies encoded within the spinal cord: evidence from focal intraspinal NMDA iontophoresis in the frog. J Neurophysiol 85: 605–619, 2001. 11160497 10.1152/jn.2001.85.2.605

[B60] Santello M, Flanders M, Soechting JF Postural hand synergies for tool use. J Neurosci 18: 10105–10115, 1998. 9822764 10.1523/JNEUROSCI.18-23-10105.1998 PMC6793309

[B61] Sasaki S, Isa T, Pettersson LG, Alstermark B, Naito K, Yoshimura K, Seki K, Ohki Y Dexterous finger movements in primate without monosynaptic corticomotoneuronal excitation. J Neurophysiol 92: 3142–3147, 2004. doi:10.1152/jn.00342.2004. 15175371

[B62] Soteropoulos DS, Williams ER, Baker SN Cells in the monkey ponto-medullary reticular formation modulate their activity with slow finger movements. J Physiol 590: 4011–4027, 2012. doi:10.1113/jphysiol.2011.225169. 22641776 PMC3476645

[B63] Takei T, Seki K Spinal interneurons facilitate coactivation of hand muscles during a precision grip task in monkeys. J Neurosci 30: 17041–17050, 2010. doi:10.1523/JNEUROSCI.4297-10.2010. 21159974 PMC6634901

[B64] Valero-Cuevas FJ, Venkadesan M, Todorov E Structured variability of muscle activations supports the minimal intervention principle of motor control. J Neurophysiol 102: 59–68, 2009. doi:10.1152/jn.90324.2008. 19369362 PMC2712269

[B65] Xu J, Ejaz N, Hertler B, Branscheidt M, Widmer M, Faria AV, Harran MD, Cortes JC, Kim N, Celnik PA, Kitago T, Luft AR, Krakauer JW, Diedrichsen J Separable systems for recovery of finger strength and control after stroke. J Neurophysiol 118: 1151–1163, 2017. doi:10.1152/jn.00123.2017. 28566461 PMC5547267

[B66] Yanai Y, Adamit N, Harel R, Israel Z, Prut Y Connected corticospinal sites show enhanced tuning similarity at the onset of voluntary action. J Neurosci 27: 12349–12357, 2007. doi:10.1523/JNEUROSCI.3127-07.2007. 17989299 PMC6673256

[B67] Zaaimi B, Edgley SA, Soteropoulos DS, Baker SN Changes in descending motor pathway connectivity after corticospinal tract lesion in macaque monkey. Brain 135: 2277–2289, 2012. doi:10.1093/brain/aws115. 22581799 PMC3381720

